# Body composition markers are more strongly associated with type 2 diabetes than inflammatory markers—Results from the study of health in Pomerania

**DOI:** 10.1111/eci.70005

**Published:** 2025-02-08

**Authors:** Saima Bibi, Muhammad Naeem, Sabine Schipf, Martin Bahls, Marcus Dörr, Nele Friedrich, Matthias Nauck, Robin Bülow, Henry Völzke, Marcello Ricardo Paulista Markus, Till Ittermann

**Affiliations:** ^1^ Institute for Community Medicine – Department SHIP/ Clinical‐Epidemiological Research University Medicine Greifswald Greifswald Germany; ^2^ Department of Zoology University of Malakand Chakdara Dir (L) Pakistan; ^3^ German Center for Diabetes Research (DZD) Partner Site Greifswald Greifswald Germany; ^4^ Department of Internal Medicine B – Cardiology, Intensive Care, Pulmonary Medicine and Infectious Diseases University Medicine Greifswald Greifswald Germany; ^5^ German Centre for Cardiovascular Research (DZHK), Partner Site Greifswald Greifswald Germany; ^6^ Institute of Clinical Chemistry and Laboratory Medicine University Medicine Greifswald Greifswald Germany; ^7^ Institute for Radiology and Neuroradiology University Medicine Greifswald Greifswald Germany

**Keywords:** C‐reactive protein, inflammation, magnetic resonance imaging, obesity, type 2 diabetes, waist circumference

## Abstract

**Background and Aims:**

Evidence links body composition and inflammatory markers with type 2 diabetes (T2D). However, the comparative analysis of body composition markers derived from different modalities and inflammatory markers in relation to T2D remains unexplored. This study aims to evaluate and compare the association of body composition and inflammatory markers with T2D.

**Methods:**

We included 4043 participants (2081 female, 51.4%) aged 20–84 enrolled in the population‐based Study of Health in Pomerania. Multivariable logistic regression models adjusted for confounding were used to analyse associations of standardized body composition markers derived from classic anthropometry, bioelectrical impedance analysis, magnetic resonance imaging as well as inflammatory markers C‐reactive protein, white blood cell count, fibrinogen, ferritin and CRP‐to‐albumin ratio with prevalent T2D.

**Results:**

Body composition markers were more strongly associated with T2D than inflammatory markers. Waist circumference exhibited the strongest association with T2D (female: odds ratio (OR) = 2.55; 95% confidence interval [CI]: 2.17–3.00; male: OR = 2.20; 95% CI: 1.86–2.60). Similarly, body weight (female: 2.07; 1.78‐2.41; male: OR = 1.99; 95% CI = 1.71–2.31), waist‐to‐height ratio (female: OR = 2.39; 95% CI = 2.05–2.77; male: 2.28; 1.92–2.70) and visceral adipose tissue (female: 3.02; 95% CI = 2.11–4.32; male: 1.50; 1.19–1.89) showed strong associations with T2D. Among inflammatory markers, white blood cell count in male and CRP‐to‐albumin ratio in female exhibit the strongest association with T2D.

**Conclusions:**

Body composition markers seem to be more tightly associated with prevalent T2D compared to inflammatory markers.

## Graphical abstract

This cross‐sectional, population‐based study aims to evaluate and compare the association of body composition and inflammatory markers with type 2 diabetes (T2D). Data from 4043 participants (51.4% women, aged 20–84 years) were analysed using multivariable logistic regression models. Results indicated that body composition markers were more strongly associated with prevalent T2D than inflammatory markers. Additionally, the findings suggest a sex‐specific role of body composition and inflammatory markers in T2D development.

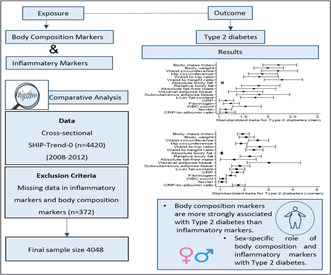

## 1 INTRODUCTION

Type 2 diabetes (T2D) is the most common metabolic disorder and one of the leading cause of many morbidities and mortality worldwide.^1^, ^2^, ^3^ T2D can affect tissues and organ functions by triggering pathophysiological pathways like inflammation, protein kinase isoforms, oxidative stress, apoptosis and transcription factors.^4^, ^5^ These pathways further lead to nephropathy, retinopathy, neuropathy, atherosclerosis, dementia and cardiovascular diseases.^6^, ^7^, ^8^ Globally, 451 million persons aged 18–99 were estimated to have diabetes in 2017 and these numbers are predicted to rise up to 693 million by 2045.^9^ Additionally, 12% of global health expenditures are attributable to the financial burden of diabetes.^10^

While several factors contribute to a higher risk of developing T2D, obesity is considered one of the most important of them.^11^, ^12^, ^13^ According to a meta‐analysis of 18 studies, the risk of T2D is seven times higher in person with obesity compared to those with normal weight.^14^ Numerous studies have investigated body composition markers to predict the risk of T2D.^15^, ^16^ A meta‐analysis and previous large‐scale studies showed that body mass index (BMI) and waist circumference (WC) have consistently strong associations with T2D.^17^, ^18^, ^19^, ^20^, ^21^ Other studies highlighted that WC, waist‐hip ratio (WHR) and waist‐to‐height ratio (WHtR)^22^, ^23^, ^24^, ^25^, ^26^ are the best anthropometric markers for predicting the risk of T2D. In contrast, some studies emphasize visceral adipose tissue (VAT) is the main culprit for T2D, underscoring its role in metabolic dysregulation.^27^, ^28^, ^29^ Additionally, sex‐specific analyses revealed distinct patterns of association between body composition markers and T2D.^30^, ^31^, ^32^ Differences in the association between obesity markers and T2D have been reported across sex, ethnicity and different age groups.^33^, ^34^, ^35^ Despite these findings, the association between body composition markers and T2D remains a topic of debate.

Along with obesity, chronic inflammation has been proposed as another major risk factor in the pathogenesis and progression of T2D and insulin resistance.^36^, ^37^, ^38^, ^39^ It is well documented that excessive adipose tissue plays a key role in the elevation of inflammatory cytokines,^40^ which are associated with a higher risk of T2D.^41^, ^42^ However, inflammation alone can impair insulin signalling,^43^ indirectly increase T2D risk even in the absence of obesity. Further studies emphasize that modulating the inflammatory system may be a promising therapeutic strategy against T2D.^4^, ^44^ In line with this, epidemiological studies, along with systematic reviews and meta‐analysis, demonstrated that inflammatory markers such as C‐reactive protein (CRP), interleukin, fibrinogen, white blood cell (WBC) and ferritin are risk factors associated with the development of T2D.^39^, ^45^, ^46^, ^47^, ^48^, ^49^, ^50^, ^51^, ^52^, ^53^, ^54^

Despite considerable research, previous studies investigating associations of body composition with T2D have limitations such as small sample sizes^30^, ^31^, ^45^, ^55^ and the inclusion of selected populations.^45^, ^56^ Furthermore, these studies used few anthropometric markers, which measure adiposity indirectly,^20^, ^21^, ^26^, ^57^, ^58^, ^59^, ^60^ for example, BMI cannot distinguish between muscles and fat mass and does not consider fat distribution in the body.^61^

Regarding inflammatory markers, none of the previous studies had investigated body composition and inflammatory markers with T2D in parallel. Moreover, previous studies mostly were conducted in the clinical setting with relatively small sample sizes^45^, ^52^, ^54^, ^62^ and focused on single inflammatory markers such as CRP,^50^, ^53^, ^54^ WBC^48^, ^49^ and ferritin.^47^, ^62^

Against this background, the primary aim of our study was to investigate associations of body composition markers derived from classic anthropometry (BMI, body height and weight, WC, hip circumference, WHT and WHtR), bioelectrical impedance analysis (BIA: absolute fat mass [FM], relative fat‐free mass [FFM]) and magnetic resonance imaging (MRI: VAT, subcutaneous adipose tissue [SAT] and liver fat content) as well as inflammatory markers (CRP, WBC, fibrinogen, ferritin and CRP‐to‐albumin ratio) with T2D. Secondly, we aimed to determine for each sex which body composition marker and which inflammatory marker is most strongly associated with T2D in a large cross‐sectional population‐based study.

## 2 MATERIALS AND METHODS

### 2.1 General population sample

In the present analyses, we used data from the Study of Health in Pomerania (SHIP‐TREND‐0), a population‐based study located in Northeast Germany.^63^ The study design have been described elsewhere.^64^ A random sample of 8826 adults was drawn from population registries, of which 4420 participants aged 20–84 years participated between 2008 and 2012 (response 50.1%). The study was conducted according to principles of the Declaration of Helsinki as reflected by an a priori approval of the Institutional Review Board of the University of Greifswald and written informed consent provided by the participants.

From our analyses, we excluded 372 individuals with missing data in the outcomes, exposures or confounders resulting in a final sample size of 4048 individuals. During the process of measuring liver fat, VAT and SAT using MRI, we further excluded individuals, who did not attend the MRI examinations, resulting in a study group consisting of 1798 individuals for liver fat content and 1854 individuals for examining VAT and SAT.^65^

### 2.2 Interview and physical examination

All participants underwent computer‐assisted face‐to‐face interviews, during which they provided information on sociodemographic and behavioural characteristics as well as on medical histories and medication intake. Smokers were categorized into three categories (lifetime nonsmokers, former smokers and current smokers). Individuals were classified as physically inactive if they reported less than 1 h/week of exercise during summer and winter. Participants were asked to bring all medications taken 7 days before the time of examination. Medication data were obtained online using the IDOM software (online drug database led medication assessment) and categorized according to the Anatomical Therapeutical Chemical (ATC) classification index. During the physical examination, standardized measurements of body height, body weight, WC, hip circumference and blood pressure were performed. BMI was calculated by dividing weight (kg) by the square of height (m^2^). WHR and WHtR were calculated. FM and FFM were measured by BIA using a multifrequency Nutriguard‐M device (Data Input, Pöcking, Germany) and the NUTRI4 software (Data Input, Pöcking, Germany) in participants without pacemakers. The electrodes were placed on hand, wrist, ankle and foot. The test frequency was measured at 5, 50 and 100 kHz following the manufacturer's instructions.^66^

### 2.3 Laboratory measurements

Fasting blood samples were taken from the cubital vein in the supine position. All samples were analysed in the Institute of Clinical Chemistry and Laboratory Medicine of the University Medicine Greifswald. CRP concentrations were determined in serum by high‐sensitive nephelometry on the Dimension VISTA (Siemens Healthcare Diagnostics, Eschborn, Germany). Fibrinogen concentrations were determined in citrate plasma according to Clauss using the BCS or the BCS XP system (Siemens Healthcare Diagnostics, Eschborn, Germany).

WBC was determined in EDTA whole blood samples using the Sysmex XT 2000, XE 5000 or SE 9000 analyzers (Sysmex, Kobe, Japan) or the Advia 2120i (Siemens Healthcare Diagnostics, Eschborn, Germany). Ferritin concentrations were determined by a chemiluminescent assay (Siemens Vista, TRF Flex® reagent cartridge, Siemens Healthcare Diagnostics Inc., Newark, DE, USA). CRP‐to‐albumin ratio was calculated.

### 2.4 Ascertainment of type 2 diabetes

Participants were classified as having T2D if they reported a physician's diagnosis of T2D in the interview or took hypoglycemic medication including oral hypoglycemic agents or insulin (ATC code A10) or had elevated blood glucose (fasting blood glucose ≥7.0 mmol/L or 2‐h post load glucose ≥11.1 mmol/L) or HbA1c levels (HbA1c ≥ 6.5%).

### 2.5 Magnetic resonance imaging examinations

VAT, SAT and liver fat content were determined by MRI using a 1.5‐Tesla system (Magnetom Avanto, Siemens Healthcare AG, Erlangen, Germany, software version syngo MR B15). Abdominal fat was determined by axial 3D datasets using the 2‐point Dixon technique (matrix: 256 × 176; slice thickness 4 mm/4 mm/3 mm without gap; 3 × 64 slices; inphase: TE 4.76 ms, TR 7.48 ms; opp‐phase: TE 2.38 ms, TR 7.48 ms). The quantification of VAT and SAT was done using ATLAS (automatic tissue and labeling analysis software), an in‐house developed software at the University of Ulm.^67^

### 2.6 Statistical methods

Continuous data was reported as median, 25th and 75th percentiles and categorical data as absolute numbers and percentages stratified by sex and WC split into two groups by the sex‐specific median. Multivariable logistic regression analyses were used for the association of body composition and inflammatory markers with T2D by calculating odds ratios (OR) and 95% confidence intervals (95% CI). All models were adjusted for age, sex, smoking status and physical inactivity. Models for FFM was adjusted additionally for absolute FM. To evaluate whether the associations of body composition (BMI and WC) and inflammatory markers (CRP and WBC) remain significant with T2D, we put the body composition and inflammatory markers into a regression model in parallel. To make the regression models comparable, all exposure variables were used as standardized variables. A *p*‐value of *p* < .05 was considered statistically significant. All statistical analyses were performed using Stata 14.2 (Stata Corporation, College Station, TX, USA).

## 3 RESULTS

Tables 1 and 2 show the characteristics of the study population stratified by WC into groups of lower median (below/equal the 50th percentile) and higher median (above the 50th percentile) for male and women. Participants in the higher median group had higher median values of body weight, WC, hip circumference, WHR, WHtR, relative body fat, FFM, VAT, SAT, liver fat content, CRP, fibrinogen, WBC, ferritin and CRP‐to‐albumin ratio and comprised a higher percentage of individuals with T2D compared to individuals in the lower median group.

**TABLE 1:** Characteristics of the study population stratified by sex‐specific percentiles of waist circumference (male).

Waist circumference		
Variable	Below 50th percentile (*n* = 989)	Above/equal 50th percentile (*n* = 978)
Body mass index in kg/m^2^	25.5 (23.7; 27.0)	31.1 (29.3; 33.5)
Body weight in kg	79.1 (73.2; 85.6)	96.5 (89.5; 105)
Waist circumference in cm	88.2 (83.4; 92.3)	106 (101; 111)
Hip circumference in cm	96.7 (93.0; 100)	106 (103; 111)
Waist‐to‐height ratio	.49 (.47; .52)	.60 (.57; .64)
Waist‐to‐hip ratio	.91 (.87; .94)	.99 (.96; 1.03)
Absolute body fat in kg	.69 (.56; .83)	1.08 (.93; 1.27)
Relative body fat in %	20.9 (17.9; 23.9)	26.9 (24.1; 30.0)
Absolute fat‐free mass in kg	62.6 (58.1; 67.2)	70.8 (66.2; 75.9)
Visceral adipose tissue in L	3.42 (2.24; 4.80)	7.25 (5.94; 8.76)
Subcutaneous adipose tissue in L	5.10 (4.06; 6.33)	8.60 (7.05; 10.3)
Liver fat content in %	3.20 (2.21; 5.28)	8.39 (4.85; 14.5)
CRP in mg/L	.81 (.43; 1.57)	1.63 (.90; 3.14)
Fibrinogen in g/L	2.60 (2.30; 3.20)	3.10 (2.50; 3.50)
White blood count in Gpt/L	5.58 (4.71; 6.70)	6.10 (5.08; 7.27)
Ferritin in μg/L	114 (66.0; 187)	176 (100; 279)
CRP‐to‐albumin ratio	.02 (.01; .04)	.04 (.02; .08)
Type 2 diabetes in %	6.09	22.21

*Note*: Data are expressed as median, 25th and 75th percentile (continuous data) or as percentage (categorical data).

Abbreviation: CRP, C‐reactive protein.

**TABLE 2:** Characteristics of the study population stratified by sex‐specific percentiles of waist circumference (female).

Waist circumference		
Variable	Below 50th percentile (*n* = 1054)	Above/equal 50th percentile (*n* = 1027)
Body mass index in kg/m^2^	23.5 (21.7; 25.4)	30.8 (28.3; 34.2)
Body weight in kg	63.8 (58.1; 68.9)	81.7 (74.6; 91.7)
Waist circumference in cm	74.9 (70.6; 79.0)	94.0 (88.3; 102)
Hip circumference in cm	94.1 (90.2; 98.5)	110 (105; 117)
Waist to height ratio	.45 (.43; .48)	.58 (.54; .63)
Waist to hip ratio	.79 (.76; .82)	.86 (.83; .89)
Absolute body fat in kg	.78 (.65; .93)	1.32 (1.12; 1.55)
Relative body fat in %	29.4 (25.9; 33.0)	38.9 (36.0; 42.1)
Absolute fat‐free mass in kg	44.9 (42.1; 47.7)	50.2 (46.7; 54.4)
Visceral adipose tissue in L	1.45 (.86; 2.25)	4.13 (3.18; 5.23)
Subcutaneous adipose tissue in L	6.51 (5.18; 7.72)	11.3 (9.84; 13.7)
Liver fat content in %	2.40 (1.88; 3.52)	6.38 (3.35; 11.7)
CRP in mg/L	.94 (.48; 2.07)	2.20 (1.12; 4.66)
Fibrinogen in g/L	2.90 (2.40; 3.30)	75.0 (37.0; 133)
White blood count in Gpt/L	5.59 (4.71; 6.78)	6.10 (5.21; 7.20)
Ferritin in μg/L	44.0 (22.0; 79.0)	75.0 (37.0; 133)
CRP‐to‐albumin ratio	.02 (.01; .05)	.06 (.03; .12)
Type 2 diabetes in %	3.23	18.11

*Note*: Data are expressed as median, 25th and 75th percentile (continuous data) or as percentage (categorical data).

Abbreviation: CRP, C‐reactive protein.

**FIGURE 1:**
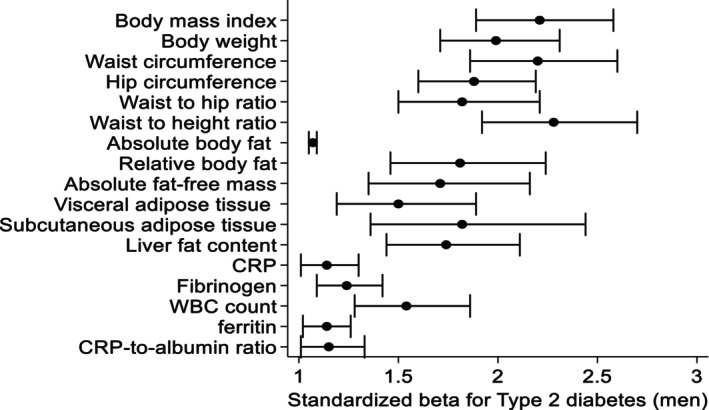
Associations between standardized body composition and inflammatory markers with type 2 diabetes in male, models adjusted for age, smoking status and physical activity and model for fat‐free mass is further adjusted for absolute fat mass. Data are expressed as standardized beta coefficient and 95% confidence interval.

**FIGURE 2:**
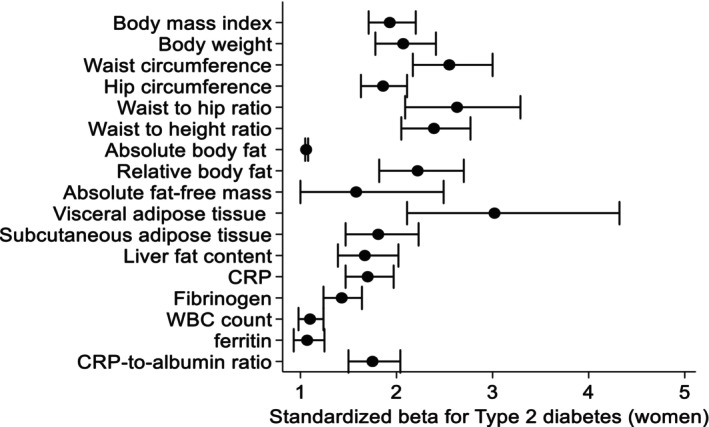
Associations between standardized body composition and inflammatory markers with type 2 diabetes in female, models adjusted for age, smoking status and physical activity and model for fat‐free mass is further adjusted for absolute fat mass. Data are expressed as standardized beta coefficient and 95% confidence interval.

In sex‐stratified logistic regression models adjusted for age, smoking and physical activity, we demonstrated significant associations of body composition and inflammatory markers with prevalent T2D (Table 3, Figures 1 and 2). Overall, anthropometric markers were more strongly associated with T2D than inflammatory markers. WC was the strongest marker for prevalent T2D in both male and female. Specifically, a one standard deviation higher WC was associated with a 155% higher chance for T2D. VAT was the body composition marker most strongly associated with T2D in female. A one standard deviation higher VAT was associated with a 202% higher chance for a prevalent T2D in female, but just with a 50% higher chance in male. Among the inflammatory markers, WBC was most strongly associated with T2D in male, while in female CRP and CRP‐to‐albumin ratio showed the strongest association with T2D.

**TABLE 3:** Odds ratios ([OR] 95% confidence interval [CI]) for the associations between standardized body composition and inflammatory markers with T2D.

	Standard deviation	Type 2 diabetes	
		Male	Female
		Odd ratio (95% CI)	Odd ratio (95% CI)
Body composition markers			
Body mass index in kg/m^2^	5.20	2.21 (1.89; 2.58)*	1.93 (1.71; 2.20)*
Body weight in kg	16.7	1.99 (1.71; 2.31)*	2.07 (1.78;2.41)*
Waist circumference in cm	14.5	2.20 (1.86; 2.60)*	2.55 (2.17; 3.00)*
Hip circumference in cm	10.7	1.88 (1.60; 2.19)*	1.86 (1.63; 2.11)*
Waist‐to‐hip ratio	.09	1.82 (1.50; 2.21)*	2.63 (2.09; 3.29)*
Waist‐to‐height ratio	.09	2.28 (1.92; 2.70)*	2.39 (2.05; 2.77)*
Absolute body fat in kg	9.54	1.07 (1.05; 1.09)*	1.06 (1.05; 1.08)*
Relative body fat in %	8.14	1.81 (1.46; 2.24)*	2.22 (1.82; 2.70)*
Absolute fat‐free mass in kg	12.1	1.71 (1.35; 2.16)*	1.58 (1.00; 2.49)*
Visceral adipose tissue in L	2.68	1.50 (1.19; 1.89)*	3.02 (2.11; 4.32)*
Subcutaneous adipose tissue in L	3.46	1.82 (1.36; 2.44)*	1.81 (1.47; 2.23)*
Liver fat content in %	6.52	1.74 (1.44; 2.11)*	1.67 (1.39; 2.02)*
Inflammatory markers			
CRP in mg/L	4.74	1.14 (1.01; 1.30)*	1.70 (1.47; 1.97)*
Fibrinogen in g/L	.74	1.24 (1.09; 1.42)*	1.43 (1.24; 1.64)*
White blood cell count in Gpt/L	2.62	1.54 (1.28; 1.86)*	1.10 (.98; 1.24)
Ferritin in μg/L	154	1.14 (1.02; 1.26)*	1.07 (.93; 1.25)
CRP‐to‐albumin ratio	.14	1.15 (1.01; 1.33)*	1.75 (1.50; 2.04)*

*Note*: Multivariable logistic regression models adjusted for age, smoking status and physical activity by calculating odd ratio. Models with fat‐free mass was further adjusted for absolute fat mass.

Abbreviations: CI, confidence interval; CRP, C‐reactive protein.

* *p* < .05.

After analysing anthropometric (BMI and WC) and inflammatory markers (CRP and WBC) together in one model, the anthropometric markers showed stronger associations with T2D than the inflammatory markers in all scenarios (Table 4). BMI and WC were significantly associated with T2D in all models. In contrast, CRP was significantly associated with T2D only in female in these models, while WBC showed a significant association with T2D only in male.

**TABLE 4:** Odds ratios ([OR] 95% confidence interval [CI]) for the combined associations of standardized body composition and inflammatory markers with T2D.

Body composition + inflammatory marker	Male	Female
Body mass index; SD + CRP; SD	2.19 (1.87; 2.57)*	1.76 (1.54; 2.02)*
	1.03 (.89; 1.21)	1.33 (1.14; 1.55)*
Waist circumference; SD + CRP;SD	2.19 (1.85; 2.60)*	2.32 (1.95; 2.76)*
	1.03 (.89; 1.20)	1.29 (1.11; 1.50)*
Body mass index; SD+ White blood cell count; SD	2.17 (1.85; 2.54)*	1.93 (1.69; 2.19)*
	1.48 (1.21; 1.80)*	1.05 (.97; 1.14)
Waist circumference; SD + white blood cell count; SD	2.16 (1.82; 2.55)*	2.53 (2.15; 2.99)*
	1.47 (1.20; 1.79)*	1.05 (.97; 1.14)

*Note*: Multivariable logistic regression models adjusted for age, sex, smoking status and physical activity.

Abbreviations: CI, confidence interval; CRP, C‐reactive protein.

* *p* < .05.

## 4 DISCUSSION

In the present large cross‐sectional population‐based study, anthropometric markers showed stronger associations with T2D than inflammatory markers. Body weight, BMI, WC and WHtR exhibited a notably stronger association with T2D than the other considered anthropometric markers. We observed that most of the associations between anthropometric markers and T2D were stronger in females than in males. Inflammatory markers were also significantly associated with T2D, but these associations were not as strong as those of the anthropometric markers. Similarly, the associations of inflammatory markers with T2D differed according to sex. For instance, CRP and CRP‐to‐albumin ratio were found to have stronger associations with T2D in females compared to males. On the contrary, WBC showed stronger associations with T2D in males when compared to females.

In line with our findings, several studies and meta‐analyses also found that WC and WHtR are more strongly associated with the risk of T2D than other body composition markers.^26^, ^68^, ^69^, ^70^ Our study adds to this body of evidence by incorporating data from MRI and BIA, emphasizing the importance of central adiposity as a key determinant of T2D risk.

Previous research has demonstrated that overweighed females are at a higher risk of developing T2D compared to males.^71^, ^72^ Consistent with this, a systematic review and meta‐analysis of observational studies reported a stronger association between DXA‐derived VAT and T2D in females than in males.^73^ In our study, we used MRI to assess VAT and observed a similar strong association between VAT and T2D in females compared to males. This stronger association in females may be attributed to the high proportion of postmenopausal women in our study population, as hormonal changes during menopause are known to influence fat distribution and metabolic risk.^74^ Recent data indicated that sex hormones, particularly oestrogen, play a pivotal role in mediating VAT and that a deficiency in these hormones may result in the aberration of adipose tissue function, which strongly contribute to the disruption of glucose metabolism and the onset of T2D.^75^ Another possible underlying mechanism for this association may be the release of free fatty acids by VAT storage, which leads to changes in insulin metabolism.^76^ An increased amount of free fatty acids can promote hepatic insulin resistance, mainly by enhancing gluconeogenesis.^77^

Moreover, we found that the inflammatory markers CRP, CRP‐to‐albumin ratio, fibrinogen and ferritin were associated with T2D, which is in line with results from a meta‐analysis and previous epidemiological studies.^45^, ^50^, ^78^, ^79^ In contrast, previous cohort studies found a positive association between CRP and increased risk of undiagnosed T2D but not incident T2D suggesting that CRP might not be a causal factor for T2D.^54^ In our study, CRP and CRP‐to‐albumin ratio were strongly associated with T2D and the association is stronger in women compared to men. One potential explanation could be that CRP is more closely associated with body fat and other metabolic parameters in female when compared to male.^80^ This suggest that low‐grade inflammation might have a larger impact on insulin levels in female. Numerous investigations have support the notion that subclinical inflammation might be correlated with insulin resistance and precede to the onset of T2D.^81^, ^82^

Additionally, we found strong associations of WBC with T2D in males, which is in accordance with a previous meta‐analysis that includes data from 20 cross‐sectional and prospective cohort studies.^83^ However, our analyses reveals sex‐specific differences in the associations of WBC with T2D. Based on our study, WBC is a more sensitive and specific marker for T2D particularly in men than in women.

### 4.1 Limitations and strengths

A limitation of our study was the cross‐sectional design, which does not allow us to draw causal inference. Thus, inflammatory processes may contribute to the development of T2D, but may be further exerted once T2D is manifest. Second, T2D assessment was based on self‐reports on a physician's diagnosis and HbA1c rather than on diagnostic procedures such as an oral glucose tolerance test. Despite adjustment for some important covariables in our analyses, one cannot exclude the possibility of residual confounding in our study. BIA was used for assessment of FM and FFM rather than dual‐energy X‐ray absorptiometry (DXA), which is the most accurate, cost‐effective and noninvasive method.^84^

One major strength of our study is the inclusion of the large sample size recruited from a general adult population. Another strength is the use of MRI for the assessment of liver fat content, VAT and SAT, which is a more reliable method than ultrasound and computerized tomography.^85^ Furthermore, we included a large number of body composition markers derived from anthropometry, BIA and MRI as well as several inflammatory markers including CRP, fibrinogen, WBC, ferritin and CRP‐to‐albumin ratio, which are fairly inexpensive and reliable inflammatory markers frequently used in clinical routine.

## 5 CONCLUSION

Our analysis provided evidence that body composition markers are more strongly associated with T2D than inflammatory markers in a general population in Germany. BMI, body weight, WC and WHtR showed the strongest association with prevalent T2D in both males and females. Our results also suggest that there is a sex‐specific role of body composition and inflammatory markers in the development of T2D and that sex‐specific prevention measures are necessary in future. Further longitudinal studies with large data sets could be a valuable addition to clarify association and explore intervention effectiveness.

## AUTHOR CONTRIBUTIONS

The manuscript was authorized by; Saima Bibi: Data analysis, manuscript writing. Muhammad Naeem, Marcello RP Markus and Till Ittermann: Data analysis and manuscript drafting. Sabine Schipf: Manuscript drafting. Martin Bahls, Marcus Dörr, Matthias Nauck and Henry Völzke: Design of the study and manuscript drafting. Nele Friedrich and Robin Bülow: Design of the study. All authors reviewed and approved the final version of the manuscript and agree to be accountable for all aspects of the work.

## CONFLICT OF INTEREST STATEMENT

The authors declare that there is no conflict of interest.

## Data Availability

Data from the ‘Study of Health of Pomerania’ are available from the University Medicine Greifswald, Germany but restrictions apply to the availability of these data, which were used under licence for the current study, and so are not publicly available. Data are, however, available upon reasonable request at https://transfer.ship‐med.unigreifswald.de/FAIRequest/ and with permission of the University Medicine Greifswald.
